# The Coordination of Local Translation, Membranous Organelle Trafficking, and Synaptic Plasticity in Neurons

**DOI:** 10.3389/fcell.2021.711446

**Published:** 2021-07-14

**Authors:** Dipen Rajgor, Theresa M. Welle, Katharine R. Smith

**Affiliations:** Department of Pharmacology, University of Colorado School of Medicine, Aurora, CO, United States

**Keywords:** local translation, synaptic plasticity, endosomes, lysosomes, neurotransmitter receptors, LTP, LTD, mitochondria

## Abstract

Neurons are highly complex polarized cells, displaying an extraordinary degree of spatial compartmentalization. At presynaptic and postsynaptic sites, far from the cell body, local protein synthesis is utilized to continually modify the synaptic proteome, enabling rapid changes in protein production to support synaptic function. Synapses undergo diverse forms of plasticity, resulting in long-term, persistent changes in synapse strength, which are paramount for learning, memory, and cognition. It is now well-established that local translation of numerous synaptic proteins is essential for many forms of synaptic plasticity, and much work has gone into deciphering the strategies that neurons use to regulate activity-dependent protein synthesis. Recent studies have pointed to a coordination of the local mRNA translation required for synaptic plasticity and the trafficking of membranous organelles in neurons. This includes the co-trafficking of RNAs to their site of action using endosome/lysosome “transports,” the regulation of activity-dependent translation at synapses, and the role of mitochondria in fueling synaptic translation. Here, we review our current understanding of these mechanisms that impact local translation during synaptic plasticity, providing an overview of these novel and nuanced regulatory processes involving membranous organelles in neurons.

## Introduction

**FIGURE 1:**
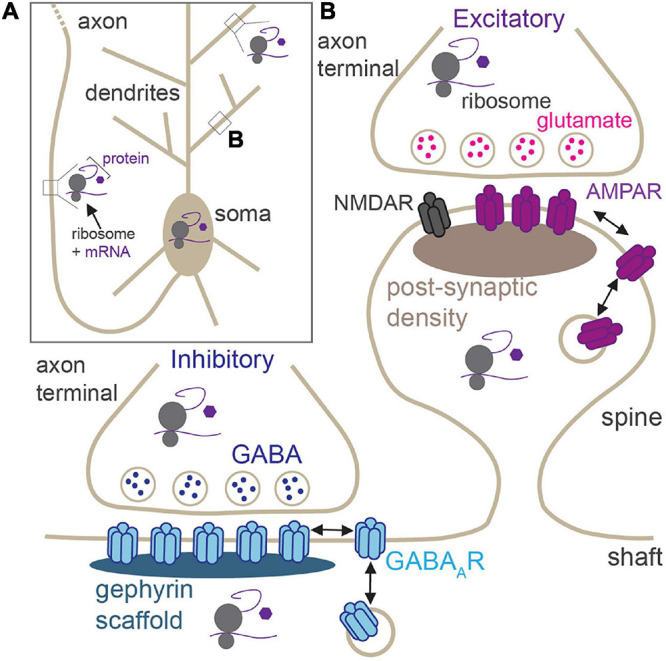
Locations of activity-dependent translation in neurons. **(A)** Neurons have elaborate, long processes (dendrites and axons) which project from the soma. Translation of synaptic proteins takes place in the soma, proximal and distal dendrites, and within the axon. **(B)** Zoom of boxed region in panel **(A)** showing excitatory and inhibitory synapses. Excitatory synapses are housed in dendritic spines and contain post-synaptic glutamate receptors (AMPAR and NMDARs). GABAergic synapses are primarily located on dendritic shafts and contain synaptic GABA_A_Rs. Local activity-dependent translation supports synaptic plasticity at both synapses in pre- and post-synaptic compartments.

Neurons are morphologically elaborate and complex cells with long and intricately branched dendrites and axons, providing spatial compartmentalization that supports their primary functions in neurotransmission and circuit connectivity. Neuronal dendrites can be millimeters in length, whereas axons can reach lengths of up to a meter in humans (Figure 1A), thereby generating an expansive network of neurites that can accommodate several thousands of synapses. These synapses can be excitatory (glutamatergic) or inhibitory (GABAergic), and each exhibits its own unique structure, function, synaptic proteome, and mechanisms of synaptic plasticity (Figure 1B; Citri and Malenka, 2008; Smith and Kittler, 2010; Henley and Wilkinson, 2016; Chiu et al., 2019; Oh and Smith, 2019). Activity-dependent modification of synaptic efficacy is a crucial mechanism at the heart of the ability of the brain to encode and retain information. Persistent changes to excitatory and inhibitory synaptic strength can be elicited by an array of processes including long-term potentiation (LTP), long-term depression (LTD), and homeostatic scaling in response to chronic changes in activity, all of which require synaptic insertion of newly synthesized proteins or alterations to synaptic protein composition at synaptic sites (Sutton and Schuman, 2006). Indeed, synthesis of new proteins is required for many diverse forms of long-lasting synaptic plasticity, learning, memory, and behavior (Kang and Schuman, 1996; Martin et al., 1997; Huber et al., 2000; Miller et al., 2002; Bradshaw et al., 2003; Sutton et al., 2006). The synaptic proteome is a key indicator of the type and strength of the synapse, and its regulation by constant protein synthesis and degradation drives synaptic homeostasis and plasticity (Bingol and Schuman, 2006; Hanus and Schuman, 2013; Dorrbaum et al., 2018). It is now well-appreciated that, given their vast size and high degree of spatial compartmentalization, neurons de-centralize protein synthesis from the soma, to locally support essential processes in remote compartments (e.g., synaptic transmission; Figure 1A). This enables rapid protein production at specific presynaptic or postsynaptic sites, or within broader regions of distal dendrites/axons, in response to synaptic activation.

In recent years, multiple studies have identified points of convergence between activity-dependent local translation at synapses and the dynamics or function of membranous organelles such as endosomes, the endoplasmic reticulum (ER), and mitochondria. As in other cell types, these organelles have canonical roles in neuronal dendrites and axons, and at synapses, producing new proteins, trafficking them to their sites of action, and fueling synaptic transmission. However, emerging evidence suggests that transport of the required RNAs to synapses can be driven by endosomes and lysosomes, activity-dependent local translation can be dynamically regulated by the endosomal system and ER, and synaptic mitochondria are tethered near synapses to fuel translation. Here, we review the latest findings that support the interplay between activity-dependent local translation and organelle transport in the context of synaptic plasticity. We will first outline the general principles of activity-dependent local translation at synapses, and then we place these novel roles for membranous organelles within this broader context.

## Activity-Dependent Translation at Synapses

mRNA localization and translation in distal neuronal dendrites and axons offer significant advantages over long-range trafficking of mature proteins from the cell soma, namely, *speed*, *specificity*, and *energy efficiency*. Indeed, modeling studies suggest that the process of delivering newly made proteins from the soma to distal and diverse synapses would be too slow and non-specific and an energy-inefficient process (Williams et al., 2016). On-demand protein synthesis from mRNAs localized near synapses overcomes this problem and rapidly produces new synaptic constituents that can enhance or reduce synaptic efficacy (Fonkeu et al., 2019). This needs to happen particularly quickly for synaptic plasticity processes, where neurons must respond rapidly to neural stimulation; in some cases, plasticity proteins are produced within minutes of stimulation (Ouyang et al., 1999). Local translation at synapses also provides synaptic specificity, which is crucial to enable neurons to fine-tune synaptic strength in response to local signaling events. A good example of this is during LTP, which is input-specific: a single synapse or a group of synapses is potentiated, whereas their neighbors are unaffected (Andersen et al., 1977; Govindarajan et al., 2011). It is therefore imperative to induce translation of the required proteins at the activated, and not the nearby inactivated, synapses to avoid aberrant, non-specific synaptic potentiation.

Lastly, local translation of synaptic proteins is energy efficient: many protein copies can be efficiently generated from a single mRNA *in situ*, as compared with the predicted energy expenditure in the transport of newly synthesized proteins from the soma to distal synapses (Schnitzer and Block, 1997; Rangaraju et al., 2019). It is now well-established that protein synthesis is a common feature of presynaptic and postsynaptic sites (Hafner et al., 2019). Translational components such as mRNAs, ribosomes, and translational regulatory elements have been demonstrated to be present in both dendritic and axonal compartments (Cajigas et al., 2012; Younts et al., 2016; Scarnati et al., 2018; Hafner et al., 2019; Biever et al., 2020). Moreover, use of recently developed techniques such as puromycin proximity ligation assays (puro-PLA; tom Dieck et al., 2015) and advanced imaging probes (Morisaki et al., 2016; Wang et al., 2016a) have enabled the visualization of translation in these compartments. In the following sections, we will provide an overview of local translation at synapses and describe how this process is crucial for long-term synaptic plasticity.

### Activity-Dependent Local Translation at Postsynaptic Sites

Local translation of proteins in postsynaptic compartments is required for numerous forms of synaptic plasticity, including LTP (Nayak et al., 1998; Miller et al., 2002), some forms of LTD (Huber et al., 2000), and homeostatic synapse scaling during periods of low or enhanced neuronal activity (Ju et al., 2004; Sutton et al., 2006; Schanzenbacher et al., 2016, 2018; Jones et al., 2018). Numerous mRNAs encoding synaptic proteins are abundant in neuronal dendrites (Cajigas et al., 2012), and recent work has provided evidence of their local translation at synaptic sites (Biever et al., 2020). Key synaptic plasticity proteins such as CaMKII and PSD95 are well documented to undergo activity-dependent translation at glutamatergic postsynaptic sites (Bagni et al., 2000; Aakalu et al., 2001; Todd et al., 2003; Muddashetty et al., 2007). CaMKII is a crucial kinase that is expressed at high levels in the brain, enriched in dendritic spines, and required for both LTP and LTD (Bayer and Schulman, 2019). Following LTP stimulation, CaMKII moves into the spine where it performs a number of crucial functions, enabling synaptic potentiation to proceed (Bayer and Schulman, 2019). Newly synthesized CaMKII can be detected in dendrites within 5 min of LTP induction (Ouyang et al., 1999; Donlin-Asp et al., 2021). Preventing dendritic localization of CaMKII mRNA in *Drosophila* impairs synaptic function and plasticity (Kuklin et al., 2017). Furthermore, disrupted CaMKII mRNA dendritic localization impacts CaMKII protein abundance in synapses, reduces late-phase LTP, and results in defects in memory behavioral tasks in mice (Miller et al., 2002), underscoring the importance of CaMKII local protein synthesis for synaptic plasticity.

In addition to the local translation of cytoplasmic signaling and scaffolding molecules, glutamate, and GABA_A_ receptors (GABA_A_Rs) are also locally translated in dendrites following synaptic stimulation (Figure 1B; Nayak et al., 1998; Ju et al., 2004; Smith et al., 2005; Sutton et al., 2006; Muddashetty et al., 2007; Rajgor et al., 2020). The abundance and composition of these neurotransmitter receptors at synapses are a major determinant underlying synaptic strength, and their trafficking to and from synaptic sites underlies changes in synaptic strength during many forms of plasticity (Malinow and Malenka, 2002; Luscher et al., 2011; Henley and Wilkinson, 2016; Chiu et al., 2019). There is ample evidence supporting the local translation of AMPA-type glutamate receptor (AMPAR) subunits in response to multiple modes of plasticity stimulation. Homeostatic compensatory scaling-up of excitatory synapses in response to chronic activity blockade involves the local dendritic synthesis of GluA1 AMPAR subunits that are incorporated into synapses to produce a receptor complement that has increased Ca^2+^-permeability and channel conductance, thus augmenting synapse function (Ju et al., 2004; Sutton et al., 2006). LTP requires protein synthesis: blocking protein synthesis following LTP induction reduces increased synaptic activity back to baseline levels (Frey and Morris, 1997). Insertion of new AMPARs into the synapse is a firm requirement for the expression of LTP (Malinow and Malenka, 2002). In line with this, local, *de novo* synthesis of AMPARs is also required to drive this process (Nayak et al., 1998). Conversely, during metabotropic glutamate receptor (mGluR) LTD, the local synthesis and trafficking of new GluA2 subunits to synapses are required, again changing the AMPAR subunit composition at synapses and inducing long-lasting changes in synaptic efficacy (Mameli et al., 2007). In addition to AMPARs, mRNAs encoding NMDA-type glutamate receptors (NMDARs) are also localized to dendrites (Cajigas et al., 2012; Udagawa et al., 2012), and the GluN2A subunit is locally translated following LTP stimulation, allowing for insertion of new NMDARs into the synapse (Swanger and Bassell, 2013).

Most work has focused on the role of local translation at excitatory synapses: posttranscriptional mechanisms underlying the translation of GABA_A_R mRNAs at inhibitory postsynapses have received far less attention (Schieweck and Kiebler, 2019). Our lab recently provided the first evidence of translation of endogenous GABA_A_R subunits in dendrites close to inhibitory postsynaptic sites (Rajgor et al., 2020). Activity-dependent local synthesis of α1 and γ2 synaptic GABA_A_R subunits was found to support inhibitory synaptic potentiation, specifically in dendrites following a form of inhibitory LTP (iLTP) induced by NMDAR activation. Multiple mRNAs encoding other GABA_A_R subunits and inhibitory synaptic proteins are also present within dendrites (Cajigas et al., 2012), although the molecular players involved in their dendritic localization and translational control remain unknown. Several RNA-binding proteins (RBPs) have been proposed by CLIP experiments to potentially facilitate their trafficking and translation (Schieweck and Kiebler, 2019). However, much further work is required to elucidate the specific mechanisms that regulate the local translation of inhibitory synaptic proteins during synaptic plasticity.

Integral membrane proteins, such as the neurotransmitter receptors mentioned above, were originally thought to be translated and assembled in the soma, trafficked through the secretory pathway, and then transported down dendrites to distal synapses. Indeed, numerous mRNAs encoding plasticity-associated synaptic integral membrane proteins, including neurotransmitter receptors, are localized in dendrites (Cajigas et al., 2012), where it is assumed that they are translated, processed, and trafficked to local synapses following plasticity stimulations. However, membrane proteins require extensive processing through the secretory pathway prior to their insertion at the plasma membrane and incorporation into synapses. This processing includes insertion into the ER for proper folding and trafficking through the ER–Golgi intermediate compartment (ERGIC) and to the Golgi apparatus for extensive glycosylation. ER, ER exit sites, and ERGIC transporters have been observed throughout the dendritic arbor, suggesting their optimal localization for processing newly generated proteins (Horton and Ehlers, 2003; Cui-Wang et al., 2012; Hanus et al., 2014). However, the Golgi apparatus is mainly localized to the cell body, presenting an intriguing conundrum as to how membrane proteins are glycosylated and trafficked to the plasma membrane in dendrites. To overcome this problem, dendrites contain specialized Golgi mini-compartments called “Golgi outposts” at dendritic branch points (Krijnse-Locker et al., 1995; Hanus et al., 2014; Hanus and Ehlers, 2016) and “Golgi satellites” near postsynaptic sites (Mikhaylova et al., 2016). Golgi satellites contain the glycosylation machinery to support protein maturation and confined processing and trafficking of surface-destined proteins to the dendritic membrane (Mikhaylova et al., 2016). Aligned with these observations, AMPAR GluA1 subunits and the cell adhesion molecule, neuroligin-1, have both been shown to traffic from the dendritic ER to the synapse via ERGIC and independently of the Golgi apparatus, pointing to a novel trafficking itinerary for newly generated synaptic membrane proteins (Bowen et al., 2017).

### Activity-Dependent Local Translation at Presynaptic Sites

Originally, much of our knowledge regarding activity-dependent local translation in distal neuronal compartments was focused on dendrites and postsynapses. Nonetheless, there is now a wealth of evidence for local translation in presynaptic compartments (Younts et al., 2016; Scarnati et al., 2018; Hafner et al., 2019). It has previously been demonstrated in invertebrates that presynaptic protein synthesis is a crucial mechanism for the induction and maintenance of diverse types of synaptic plasticity (Martin et al., 1997; Sherff and Carew, 1999; Beaumont et al., 2001; Zhang and Poo, 2002). In one of the earliest demonstrations of local translation in plasticity, Kandel and colleagues explored long-term facilitation (LTF) in *Aplysia* sensory-motor synapses. They found that LTF required CREB-mediated modulation of gene expression as well as translation in the presynaptic neuron. Furthermore, physical separation of the presynaptic compartments from the cell bodies illustrated that this translation occurred locally, in neuronal axons, near presynaptic sites (Martin et al., 1997).

More recent studies have cemented presynaptic translation as a requirement for long-term synaptic plasticity in mature mammalian neurons. Elegant tools such as axon-TRAP-RiboTag have enabled identification of the axonal translatome in adult mammalian neurons and revealed that axonal mRNA translation plays a significant role in maintaining synaptic function in mature retinal ganglion circuits (Shigeoka et al., 2016). Active, functional ribosomes have been localized to the presynaptic terminal of the calyx of Held nerve terminal, a large glutamatergic synapse in the auditory brain stem (Scarnati et al., 2018). This work provided the first visualization of local translation at mature presynaptic sites in brain slices and revealed that alterations to protein synthesis adjusted both spontaneous and evoked release (Scarnati et al., 2018). A crucial role for local protein synthesis has also been shown at GABAergic presynaptic terminals. Younts et al. (2016) utilized a small molecule, membrane-impermeable translational inhibitor, paired with electrophysiological recordings from hippocampal neurons, to reveal that LTD of inhibitory synapses requires presynaptic cap-dependent protein synthesis and is mediated by endocannabinoid receptor activation and mTOR signaling. Expanding on these studies, a recent study visualized newly synthesized proteins in excitatory and inhibitory presynaptic and postsynaptic compartments using expansion microscopy (Hafner et al., 2019). In agreement with previous work, the authors showed that presynaptic terminals (and postsynapses) in hippocampal and cortical brain sections are translationally competent. Importantly, regulation of translation was compartment-specific in response to different forms of plasticity; potentiating treatments with neurotrophins induced translation at excitatory and inhibitory presynapses, while depression via mGluR and endocannabinoid receptor activation stimulated translation in postsynapses and inhibitory presynapses, respectively (Hafner et al., 2019). Studies like these add to a nuanced model of plasticity, in which local translation can contribute both presynaptically and postsynaptically in order to alter the strength of communication under different forms of activity-dependent modulation.

## RNA Trafficking to Remote Postsynapses and Axon Terminals

Local translation is clearly imperative for proper synaptic function and plasticity, and mRNAs encoding synaptic proteins are localized to and translated at synaptic sites. But given the complexity of the neuronal neurite arbor and the sheer number of synapses formed onto any one neuron, how do mRNAs accurately reach the correct distal sites in neurons? As in other cell types, mRNAs first need to be directed to the correct neuronal compartment and then transported to their final destination. Here, we outline these mechanisms and discuss recent findings in which membranous organellar transport contributes to these processes.

### Sorting mRNAs for Localization to Distal Neuronal Compartments

What strategies do neurons use to discriminate between mRNAs that are destined to localize to the cell body or to distal sites? mRNA destination is identified through specific sequence structures within the mRNA itself, called *cis*-elements (Buckley et al., 2011; Khaladkar et al., 2013). These elements are frequently referred to as “zip codes,” which tag the mRNA with its destination and are most often, but not exclusively, found in the mRNA 3′-untranslated region (3′-UTR; Andreassi et al., 2018). mRNA *cis*-elements/zip codes are recognized by *trans*-acting RBPs, which are instrumental in directing the localization and transport of the mRNA to its final location. There are numerous RBPs that are either expressed exclusively in the brain or are ubiquitously expressed but have unique functions in the nervous system, for example, FMRP, ELAVL, and Nova proteins (Darnell, 2013). However, due to their complex and promiscuous interactions, often with multiple different mRNAs, the interactions between RBPs and their cognate *cis*-elements have been challenging to decipher (see Engel et al., 2020, for a comprehensive review).

As RBPs most often bind mRNA within the 3′-UTR, it is unsurprising that diversity in 3′-UTR sequences for the same gene can give rise to distinct mRNA localization patterns and translational regulation. Recent findings show that through the use of alternative poly-adenylation signals, genes are capable of giving rise to multiple mRNA transcripts harboring 3′-UTRs of different lengths (Miura et al., 2014; Tushev et al., 2018; Bae and Miura, 2020). Indeed, 3′-UTR length has been shown to underlie mRNA dendritic localization (Tushev et al., 2018), stability (Pickard et al., 2008), and ultimately synapse function (Kuklin et al., 2017). This is likely due to the presence/absence of regulatory regions, which harbor RBP-binding sites or microRNA (miRNA) seed sites, which allow for control of mRNA localization, translation, and stability. Interestingly, compared with other cell types, neurons have longer 3′-UTR mRNA variants for specific genes (Tushev et al., 2018), which indicate not only that neuronal mRNAs are more likely to undergo additional posttranscriptional regulation but also that additional complex mechanisms are likely to influence their spatial localization.

### Transport of mRNA to Remote Neuronal Compartments

RNAs are trafficked from their site of production in the nucleus to distal compartments via long-range transport mechanisms along dendrites and axons (see Fernandopulle et al., 2021, for a recent review). For transport, RNAs and RBPs self-assemble into macromolecular ribonucleoprotein (RNP) complexes, termed RNP granules (Formicola et al., 2019). This process of RNA granule self-assembly occurs by liquid–liquid phase separation: liquid bodies de-mix from the cytosol to form a membraneless organelle (Weber and Brangwynne, 2012; Formicola et al., 2019). Granule formation is extremely dynamic, allowing for rapid and reversible disassembly, and the release of mRNA for translation in response to extracellular cues. Biochemical characterization of RNP granule content reveals them to be highly heterogeneous and composed of diverse complements of proteins, RNAs, and ribosomal components (Kanai et al., 2004; Elvira et al., 2006; Fritzsche et al., 2013; El Fatimy et al., 2016); and this molecular diversity is thought to strongly direct granule trafficking to the axon or dendrite, spine, or shaft (Christie et al., 2009; Mitsumori et al., 2017). Further, axons and dendrites likely utilize compartment-specific mechanisms to select which RNAs are delivered to their distal regions. For example, the axon initial segment is thought to act as a filter for the selection of axon-specific cargoes and exclude those destined for dendrites (Song et al., 2009; Watanabe et al., 2012; Petersen et al., 2014).

**FIGURE 2:**
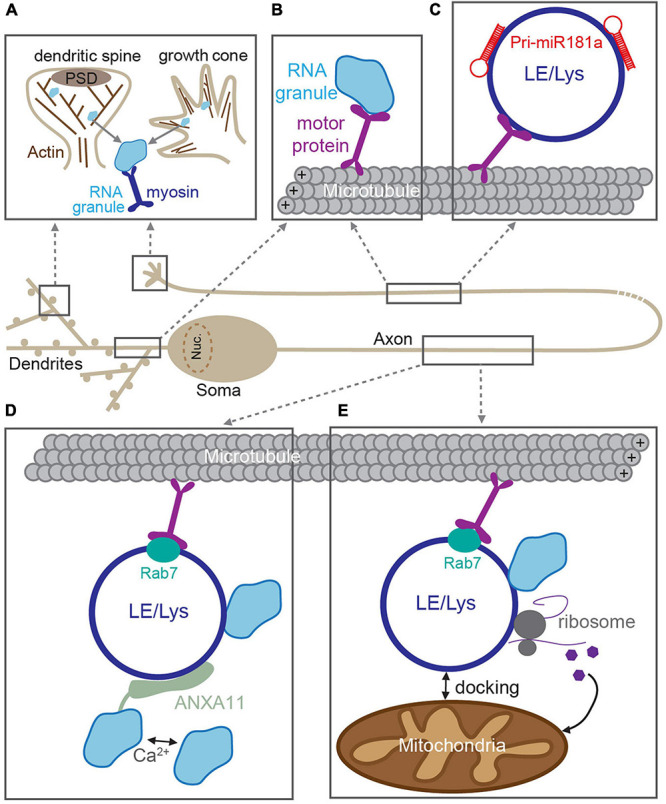
RNA trafficking along neuronal processes. RNAs are incorporated into RNA granules and transported through dendrites and axons along cytoskeletal tracks. **(A)** In dendritic spines and growth cones, RNA granules are transported along F-actin filaments by myosin motors. **(B)** In dendrites and axons, RNA granule active-transport is mediated by kinesin and dynein motors along microtubules. Transport can be mediated through direct association between RNA granules and motors. In axons, RNA granules are transported through indirect association via membranous compartments such as late endosomes or lysosomes (LE/Lys) **(C–E)**. **(C)** Pre-miRNAs are trafficked to developing growth cones via direct interactions with LE/Lys (Corradi et al., 2020). **(D)** LE/Lys act as a vesicular transporters for RNA granules. RNA granules are tethered to the rab7-positive LE/Lys via a Ca^2+^-dependent interaction with the adaptor ANXA11 (Liao et al., 2019). **(E)** Rab7-positive LE/Lys transport RNA granules and translational machinery along microtubules and can dock at mitochondria, and produce proteins required for mitochondrial function (Cioni et al., 2019).

It is well-established that RNA granules are trafficked to distal locations in dendrites and axons via active transport along cytoskeleton tracks [extensively reviewed in Dalla Costa et al. (2021), Das et al. (2019), and Maday et al. (2014)]. For long-range transport in neurites, granules are canonically transported along microtubules using the kinesin and dynein families of motor proteins. In axons, microtubules have their plus end oriented toward the distal axon, on which kinesin motors mediate anterograde transport (Kanai et al., 2004). In contrast, microtubules have mixed polarity in proximal dendrites, allowing for bidirectional kinesin-mediated transport (Das et al., 2019). RBPs within the granule can interact with the motors either directly or via adaptor proteins (Figure 2; Kanai et al., 2004; Hirokawa, 2006; Kiebler and Bassell, 2006; Davidovic et al., 2007; Dictenberg et al., 2008). Transport of mRNAs that encode the key plasticity proteins, such as CaMKII (*CaMKIIa*) and β-Actin (*ActB*), occurs via this mechanism on microtubule tracks (Tiruchinapalli et al., 2003; Mikl et al., 2011; Wu et al., 2020). In contrast, short-range trafficking of RNA granules is mediated by myosin motors on F-actin filaments (Figure 2). This mode of transport is used for the “final mile”: to transport granules into specialist, actin-rich structures such as dendritic spines or growth cones (Yoshimura et al., 2006; Mitsumori et al., 2017; Das et al., 2019).

### Long-Range RNA Transport via Endosomes/Lysosomes

Emerging evidence now suggests that long-range RNA granule trafficking can overlap with transport of late endosomes and lysosomes in a non-canonical mode of microtubule-based transport (Figure 2; Jansen et al., 2014; Salogiannis and Reck-Peterson, 2017; Pushpalatha and Besse, 2019). Compelling recent experiments from several groups indicate that RNA granules can “hitchhike” onto endosomes/lysosomes that are themselves being trafficked up and down neurites (Pushpalatha and Besse, 2019). This alternate mode of transport is thought to confer several advantages. First, vesicular trafficking is often faster than that of most cytoskeletal/soluble proteins (Maday et al., 2014), thereby making “hitchhiking” to distal locations a potentially more rapid process. Second, membranous organelles such as lysosomes provide an interaction platform for association with multiple cargoes simultaneously [e.g., with mitochondria and translational machinery (Cioni et al., 2019)], thereby creating translational super-complexes that can increase processivity. RNA granules were originally shown to traffic with endosomes to mediate long-distance movement in filamentous fungi, suggesting that this system could also be used by higher-order organisms (Baumann et al., 2012, 2014; Higuchi et al., 2014; Pohlmann et al., 2015). Indeed, a similar mechanism was then posited in neurons, in which miRNAs and the RNAi machinery were found to co-transport with acidic endosomal compartments in cultured motor neurons (Gershoni-Emek et al., 2018). More recently, live imaging of molecular beacons was elegantly utilized to detect pre-miRNAs (the precursors for mature miRNAs) in the axons of cultured *Xenopus* retinal ganglion cells (Corradi et al., 2020). These experiments revealed that pre-miR-181a is able to hitchhike onto the lipid surface of late endosomes/lysosomes for long-distance trafficking along axons (Figure 2). This enabled inactive pre-miRNAs to be delivered to axonal growth cones, where they then could be processed into mature miRNAs in response to stimulation, inhibit local translation, and guide growth cone steering (Corradi et al., 2020).

In addition to miRNAs and their precursors, mRNAs were also shown to hitchhike onto late endosomes in *Xenopus* retinal ganglion neurons (Cioni et al., 2019). RNA granules were found to associate with motile Rab7a-positive late endosomes, together with translational machinery, and mitochondria (Figure 2; Cioni et al., 2019). These membranous complexes serve as translation platforms to supply new proteins in axons, including those that will directly support mitochondrial function (Cioni et al., 2019). Interestingly, late endosomal trafficking, axonal translation, and mitochondrial integrity are disrupted by Rab7a mutations that are associated with type 2B Charcot–Marie–Tooth neuropathy (Cioni et al., 2019), underscoring that correct RNA transport and local translation are crucial for axonal function. In a complementary study, RNA granules were found to associate with moving lysosomes in rat, zebrafish, and human-derived neuronal axons (Liao et al., 2019). To identify the molecular adaptor that connected RNA granules and the lysosome, the authors used ascorbate peroxidase (APEX) proximity labeling of LAMP1-positive lysosomes, enabling the local proteome of these structures to be identified. Annexin A11 (ANXA11), an RNA granule-associated phosphoinositide binding protein, was found to tether subsets of RNA granules to lysosomes (Figure 2). The ANXA11-mediated association between RNA granules and the lysosome was required for long-range granule transport and dependent on the presence of Ca^2+^-influx and phospholipids, thereby allowing for granule release in response to rapid signaling events. Moreover, amyotrophic lateral sclerosis (ALS)-associated mutations in ANXA11 disrupted the docking of RNA granules to lysosomes and impaired RNA granule transport, suggesting that disrupted membranous RNA transport could contribute toward ALS neuropathy and pathogenesis (Liao et al., 2019).

Together, these recent studies provide strong evidence that late endosomes and lysosomes contribute to fast, long-distance RNA transport and local protein synthesis in neuronal axons, which is important for RNA delivery to synaptic sites. However, whether this mode of transport is important for dendritic RNA granule trafficking remains unclear. Given the prevalence of endosomal trafficking in dendrites, the function of mitochondria and local translation at dendritic branch points and synapses, and the critical role that these components play in synaptic plasticity, it is tempting to suggest that analogous mechanisms may also exist in dendrites. To support this notion, Liao et al. (2019) observed the LAMP1–ANXA11 association in dendrites in addition to axons, hinting that lysosomal hitchhiking for long-range transport could also exist in the dendritic arbor. Other outstanding questions include how a particular transport strategy is selected: β-actin mRNA is trafficked by both conventional trafficking mechanisms and vesicular hitchhiking (Liao et al., 2019; Wu et al., 2020), suggesting that a given mRNA could be transported by either mode of transport. Understanding under what physiological circumstances (e.g., certain types of plasticity, cell stress) these transport itineraries are selected will be imperative to fully define the process of RNA granule trafficking to synapses.

## Controlling Activity-Dependent Translation at Synaptic Sites

### RNA Granule Reorganization Induces Translation

Once an RNA granule arrives at its destination, translation begins in response to a stimulus. This process is generally reliant on stimulus-dependent changes to the RNA granule components, which release the mRNA from the granule, into the cytosol; details of this process remain to be fully characterized, however. Given their unique and dynamic structure, the ability of RNA granules to rapidly assemble, reorganize, and dissolve in response to environmental changes and external stimulation makes them ideally suited to transducing external signals in distal neuronal compartments. Depolarization of cultured neurons by KCl application induces the transition of dense granules to less compact structures, resulting in the release of mRNAs that can then be received by polysomes and can initiate translation (Krichevsky and Kosik, 2001). Activity-dependent granule reorganization is accompanied by changes in their dynamics and increased turnover (Cougot et al., 2008; Park et al., 2014). This is thought to go hand-in-hand with translational activation of synaptically localized mRNAs; experiments have shown that dissolution of granules following NMDAR stimulation is associated with the translation of dendritically localized mRNAs, including *CaMKIIa* (Zeitelhofer et al., 2008; Baez et al., 2011). Granule dissolution depends on the kind of stimulation and the distinct RBPs within the granule (Zeitelhofer et al., 2008; Baez et al., 2011; Luchelli et al., 2015).

Posttranslational modifications (PTMs) appear to have key roles in driving granule rearrangements leading to RNA release. As many PTMs are controlled by membrane receptor-driven signaling cascades from external stimulation, these modifications can link extracellular cues to changes in granule state and ultimately translation. For example, phosphorylation of RNA granules can reduce/promote granule phase separation depending on the granule composition and thereby control the release of bound mRNA (Monahan et al., 2017; Tsang et al., 2019; Ryan et al., 2021). Protein methylation by protein arginine methyltransferases has also been shown to reduce phase separation and thereby favor granule disassociation and RNA release (Qamar et al., 2018; Ryan et al., 2018; Tsang et al., 2019). In addition to PTMs, modifications to the mRNAs themselves can also trigger their translation following plasticity stimulations. For example, during LTP stimulation, mRNAs localized at synapses undergo 3′-UTR cleavage and polyadenylation, which can generate translatable forms of the mRNA (Udagawa et al., 2012; Fontes et al., 2017; Andreassi et al., 2018).

### MiRNAs Fine-Tune Local Protein Synthesis During Synaptic Plasticity

Key modulators of local protein synthesis during synaptic plasticity are miRNAs. These highly expressed, non-coding, small regulatory RNAs inhibit translation by recruiting RNA-inducing silencing complexes (RISCs) to target mRNAs through complementary base pairing in the 3′-UTR (Pratt and MacRae, 2009). MiRNAs regulate the translational events involved in development and plasticity [reviewed extensively in Hu and Li (2017); McNeill and Van Vactor (2012), and Wang et al. (2012)], and their dysfunction is implicated in numerous neurological diseases (Thomas et al., 2018). MiRNAs are expressed within the cell soma and throughout the dendrites and axons of neurons and are therefore ideally suited to regulation of localized protein synthesis at synaptic sites. Many miRNAs are enriched in synaptosomes (Xu et al., 2013) or found in close proximity to synapses (Schratt, 2009; Sambandan et al., 2017; Park et al., 2019), and their activity is tightly regulated by synaptic activity, making them ideal candidates for regulating activity-induced local translation at synapses (Rajgor and Hanley, 2016). Indeed, there are now a wealth of data to support crucial roles for numerous miRNAs in synaptic plasticity at both excitatory and inhibitory synaptic sites. Critically, multiple independent miRNA pathways converge to control dendritic spine morphology (Hu et al., 2014; Gu et al., 2015; Park et al., 2019), the trafficking of AMPARs and GABA_A_Rs, and excitatory and inhibitory synaptic transmission (Saba et al., 2012; Letellier et al., 2014; Hu et al., 2015; Olde Loohuis et al., 2015; Rajgor et al., 2020), following various forms of LTP and LTD. Several miRNAs are shown to target AMPAR mRNAs, with each miRNA responding to specific forms of synaptic activity and controlling either the upregulation or downregulation or GluA1 or GluA2 AMPAR subunit expression and synaptic incorporation (Saba et al., 2012; Hu et al., 2015; Olde Loohuis et al., 2015). At inhibitory synapses, the activity-dependent local translation of two key synaptic GABA_A_Rs (α1 and γ2) are robustly controlled in a miRNA-dependent manner, enabling the persistent maintenance of iLTP (Rajgor et al., 2020).

In many of the cases cited above, synaptic stimulation changes the expression levels of the miRNA, altering the local translation of their targets and impacting synaptic efficacy. In numerous plasticity situations, changes in miRNA-controlled mRNA translation are required, and these have been documented during several types of synaptic stimulation including LTP and LTD (Hu et al., 2014; Gu et al., 2015). However, the process of changing miRNA expression levels is slow (>30 min) and requires changes in miRNA transcription and stability. An alternative way to more rapidly increase the amount of miRNA-mediated translational repression near a synapse is to process a mature miRNA locally from its precursor miRNA (pre-miRNA). Pre-miRNAs and the machinery for their processing to mature miRNAs are expressed in axons, dendrites, and synapses (Lugli et al., 2005, 2008; Bicker et al., 2013; Sambandan et al., 2017), but it was only recently that pre-miRNA processing was shown to occur at synapses in response to synaptic activity. In a groundbreaking work, Sambandan et al. (2017) developed a novel fluorescent sensor enabling the visualization of miRNA maturation in neuronal dendrites. Using glutamate uncaging to locally stimulate specific dendritic spines, this study demonstrated rapid (within 30 min) dicer-dependent cleavage of a precursor miR-181a (pre-miR-181a) locally in spines and dendrites. This increase in pre-miRNA processing and miR-181a production resulted in a local reduction in the synthesis of its target, CaMKII. This showed that pre-miRNA processing could occur locally in dendrites and spines in response to synaptic stimulation, thereby providing mature miRNA for rapid and spatially restricted translational repression on demand.

### A Key Role for the Endoplasmic Reticulum and Endosomes in Rapid Activity-Dependent Local Translation

In addition to the mechanisms described above, a body of recent work has implicated the endosomal system and ER in the rapid modulation of local, activity-dependent translation at synapses, via the regulation of miRNA availability and activity. Similar to other cell types, the endosomal system in neurons is primarily involved in membrane protein trafficking, to and from the plasma membrane, and in the case of synaptic plasticity, regulating the abundance of neurotransmitter receptors at the synapse (Parkinson and Hanley, 2018; Kennedy and Hanus, 2019). Early, late, and recycling endosomes are all found in close proximity to synaptic sites, where they can coordinate the delivery and removal of new proteins to the synapse (Parkinson and Hanley, 2018). In addition, the ER is present throughout the dendritic arbor, enriched at branch points, and protrudes into some dendritic spines, where it can coordinate the production of newly produced proteins (Horton and Ehlers, 2003; Cui-Wang et al., 2012; Hanus et al., 2014; Kennedy and Hanus, 2019).

**FIGURE 3:**
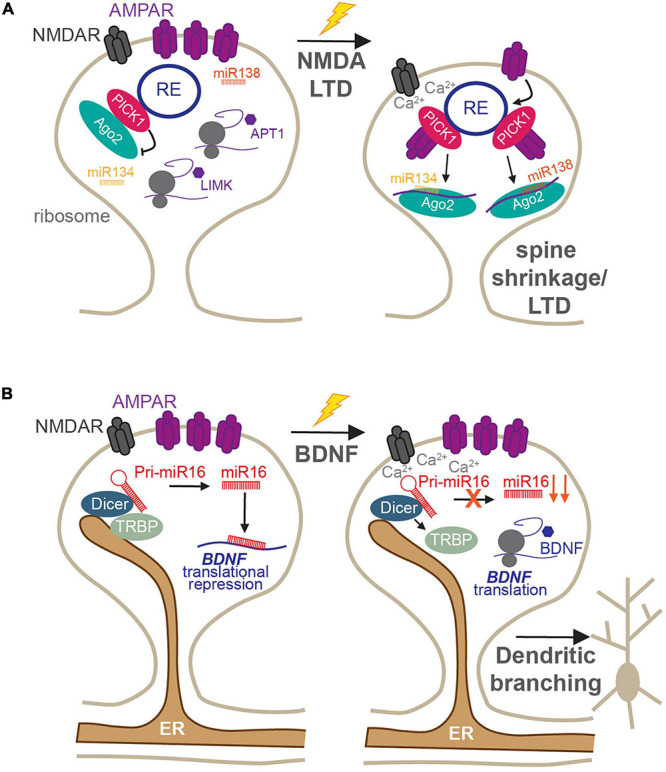
Roles of membranous organelles in the regulation of local protein synthesis at synapses. **(A)** The Ca^2+^-sensing AMPAR trafficking protein PICK1 regulates the activity of Ago2 in dendritic spines. Under basal conditions, PICK1 directly recruits Ago2 to recycling endosomes which reduces Ago2 silencing of miR134-LIMK1 and miR138-APT1 and maintains enlarged spines. Following NMDA receptor stimulation, the local rise in Ca^2+^-concentration disrupts PICK1-Ago2 interactions. This increases the affinity of PICK1 for AMPARs, enhancing their internalization, increases Ago2-mediated miR134-LIMK1 and miR138-APT1 silencing, and supports persistent LTD (Antoniou et al., 2014; Rajgor et al., 2017, 2018). **(B)** The ER is present within dendrites and spines, where it scaffolds Dicer complexes to locally control miRNA biogenesis. Under basal conditions, miR16 is processed locally to translationally repress BDNF and inhibit dendritogenesis. Following BDNF stimulation, TRBP dissociates from Dicer in a calcium-dependent manner, resulting in reduced miR16 maturation and increased translation of BDNF to increase dendritic growth (Antoniou et al., 2018).

In addition to these canonical roles, both synaptic endosomes and ER provide mechanisms by which miRNA-mediated local translation can be quickly modified in response to synaptic stimulation. The RISC machinery, which is responsible for miRNA-mediated silencing, is also associated with the endosomal system in dendritic spines (Antoniou et al., 2014), and changes in its activity have been shown to rapidly regulate specific miRNA–mRNA silencing events. The major RISC protein, Ago2, localizes to recycling endosomes in neuronal dendrites via a direct interaction with PICK1 (Figure 3A; Antoniou et al., 2014). PICK1 is a Ca^2+^-sensitive, BAR/PDZ domain protein that has essential roles in AMPAR receptor trafficking and modulation of dendritic spine morphology during LTP and LTD (Hanley, 2008; Parkinson and Hanley, 2018). Recycling endosomes are found in or at the base of dendritic spines and have crucial roles in spine growth and driving the increase in AMPAR synaptic expression during LTP (Park et al., 2006; Schmidt and Haucke, 2007; Hiester et al., 2017). Thus, the Ago2–PICK1 interaction in these compartments provides an intriguing link between the synaptic endosomal system and the local translational machinery that is responsible for rapidly altering the synaptic proteome. As a Ca^2+^-sensor, PICK1 can respond to NMDAR activation to regulate its protein–protein interactions (Hanley, 2008), and NMDAR-mediated Ca^2+^-influx during LTD disrupts the association of Ago2 with PICK1-containing recycling endosomes (Figure 3A). This enables Ago2 to specifically increase miRNA-dependent translational repression of proteins involved in maintaining the structural plasticity of dendritic spines, including LIM kinase 1 (LIMK1) and APT1, thus leading to spine shrinkage (Figure 3A; Rajgor et al., 2017). The same stimulation was also shown to transiently induce phosphorylation of Ago2 at S387 by the kinase Akt and to increase Ago2 association with the two core RISC proteins, GW182 and DDX6 (Rajgor et al., 2018). These RISCs rapidly and specifically increase translational repression of LIMK1 via miR-134 in dendrites, which is essential for the shrinkage of dendritic spines following NMDA stimulation. Interestingly, both dissociation of Ago2 from endosomes and its phosphorylation by Akt are not mutually exclusive events but are likely to represent two independent pathways that maintain long-term dendritic spine shrinkage during LTD.

The dendritic ER has recently been shown to act as a regulatory compartment for pre-miRNA processing in dendrites, by providing a platform for pre-miRNA processing complexes (Antoniou et al., 2018). The miRNA generating complex, consisting of Dicer and its cofactors, including the transactivation response element RBP (TRBP), was found to be associated with the ER in the soma and dendrites of developing neurons (Figure 3B). This association was dynamic: following brain-derived neurotrophic factor (BDNF) stimulation, TRBP dissociated from dendritic ER and Dicer in a Ca^2+^-dependent manner (Figure 3B; Antoniou et al., 2018). This reduced the processing of a subset of pre-miRNAs, including miR-16, a negative regulator of dendrite complexity and BDNF-mediated dendritogenesis. Relieving miR-16-mediated translational repression allowed the translation of its target, the mRNA encoding BDNF itself, facilitating BDNF production for its crucial roles in neuronal development and function (Antoniou et al., 2018). Given the enrichment of ER at dendritic branch points (Cui-Wang et al., 2012), this provides an elegant mechanism by which local protein synthesis could drive the branching of neurites during development, which has important ramifications for neuronal and circuit function. It will be important to determine whether similar mechanisms exist in mature neurons and whether they may play a role in pre-miRNA processing in response to synapse-specific stimulation, as shown in Sambandan et al. (2017). If so, this would place the dendritic/spine ER as a prime site for synapse-specific remodeling of the synaptic proteome, which would have fundamental importance for input-specific plasticity and dendritic integration.

Together, these studies demonstrate how synaptic signaling can power the local translation of specific mRNAs through the rapid modulation of miRNA activity, by either altering RISC-miRNA-mediated translational repression or increasing mature miRNA processing, both via interactions with endosomes or the ER. Currently, these mechanisms are only established for limited miRNA-mediated silencing events: it will be crucial to determine if these forms of translational regulation are used broadly across multiple miRNAs. Defining which synapses display these types of regulation is also critical. Indeed, only a subset of dendritic spines contain recycling endosomes or ER; this thereby limits these mechanisms to those spines, which are most often stable and mature spine types. Furthermore, it is unclear if either of these mechanisms have roles at inhibitory synapses. These synapses are located near ER and the endosomal system in the dendritic shaft; therefore, it is certainly possible that similar organelle-dependent modes of translational regulation could also contribute to GABAergic synaptic plasticity.

## Fueling Local Translation: The Role of Mitochondria

Establishing, changing, and maintaining functioning synapses consumes a significant amount of energy. To meet these energy demands, neurons must be equipped to sufficiently power synaptic processes, particularly for activity-dependent synaptic modulation. In neurons, mitochondria are critical in supporting synaptic communication, particularly by powering local translation, both under basal conditions and during plasticity (Spillane et al., 2013; Rangaraju et al., 2019). Although mitochondria play pivotal roles in neuronal development and synaptogenesis, their dense distribution in mature neurons highlights their importance for powering neuronal functions beyond the initial formation of neural circuits [reviewed in Devine and Kittler (2018), MacAskill et al. (2010), and Son and Han (2018)]. Notably, mitochondria are distributed throughout axons and dendrites and play a role in fueling local translation in neurites under basal conditions. Spillane et al. (2013) demonstrated that when mitochondrial respiration and protein synthesis are disrupted, correct axonal branching and growth, which mediate neuronal circuit development and plasticity, are prevented. Furthermore, a recent study from the Schuman lab directly demonstrated that mitochondria in neuronal dendrites provide energy for local translation. Using a combination of inhibitors to target ATP provided by mitochondrial or glycolytic sources, as well as puromycin proximity ligation to visualize newly synthesized proteins of interest, the authors established that mitochondria are the primary source of energy fueling dendritic translation during synaptic plasticity (Rangaraju et al., 2019). When synapses were stimulated by glutamate uncaging to induce long-term plasticity, local mitochondrial depletion prevented the morphological changes that accompany potentiation at nearby spines. Furthermore, these mitochondrial-depleted regions showed significantly reduced dendritic protein synthesis compared with stimulated regions with functional mitochondria (Rangaraju et al., 2019). These results establish the importance of mitochondrial function for fueling dendritic translation in order to facilitate long-term synaptic alterations in response to neuronal activity. While there has not been a similar study demonstrating a direct relationship between mitochondrial respiration and axonal translation during plasticity, mitochondrial dynamics (i.e., fission and fusion) in axons can impact presynaptic vesicle release, an integral mechanism of synaptic communication that is supported by local translation (Lewis et al., 2018). Furthermore, mitochondria can act as hotspots of translation at axonal branchpoints (Cioni et al., 2019), suggesting that they likely play a crucial role in producing the energy required for local translation in axons. Finally, while it is clear that ATP production via mitochondrial respiration is a vital energy source for translation, it is not the only one. Recent work has shown that glycogenolysis and lactate transport from astrocytes to neurons is also critical for powering protein synthesis during certain forms of plasticity and long-term memory formation (Descalzi et al., 2019). A greater understanding of how neurons accommodate increased energy demands during synaptic transmission and plasticity will provide key insights into activity-dependent modulation of neuronal communication and its dysfunction in pathology.

## Conclusion and Perspectives

It is clear that membranous organelles and vesicles are emerging as critical mediators that facilitate RNA trafficking and translation during synaptic plasticity. This represents a fascinating new avenue of research combining RNA biology, synaptic transmission/plasticity, and cell biology. We have described a number of recent studies that show membranous organelles are important for select modes of RNA trafficking, converting synaptic stimulation to changes in local gene expression, and providing an energy source and “hotspot” for local translation. As these phenomena have only been described in a limited number of situations (e.g., NMDAR-dependent LTD; Antoniou et al., 2014), a prominent focus should now be on determining how broadly utilized these mechanisms are, not only across different forms of synaptic plasticity but also across diverse synapses, within a single neuron and in alternate brain regions. For example, are miRNA processor complexes present on ER networks close to inhibitory synapses as well as in some spines? Conversely, understanding which mRNAs and/or miRNAs are regulated by these mechanisms will be a crucial step to more broadly appreciating the roles of membranous transport in controlling activity-dependent translation in neurons. Lastly, given that defects in RNA trafficking and translation are tightly linked to genetic disorders such as ALS and Fragile X syndrome (Wang et al., 2016b; Fernandopulle et al., 2021), further investigation into the role of organelle/local translation cross-talk in synaptic dysfunction will surely add to this body of knowledge. As research in this area is only in its infancy, it is likely that further investigation will reveal fundamental roles for endosomes, lysosomes, and mitochondria in activity-dependent local translation and how they are disrupted in neurological disease.

## Author Contributions

DR, TW, and KS conceptualized the review and wrote and illustrated the manuscript. All authors contributed to the article and approved the submitted version.

## Conflict of Interest

The authors declare that the research was conducted in the absence of any commercial or financial relationships that could be construed as a potential conflict of interest.
